# The miR-290-295 cluster as multi-faceted players in mouse embryonic stem cells

**DOI:** 10.1186/s13578-017-0166-2

**Published:** 2017-08-07

**Authors:** Kai Yuan, Wen-Bing Ai, Lin-Yan Wan, Xiao Tan, Jiang-Feng Wu

**Affiliations:** 1 Institute of Organ Fibrosis and Targeted Drug Delivery, Medical College, China Three Gorges University, 8 Daxue Road, Xiling District, Yichang, 443002 China; 2 The Yiling Hospital of Yichang, 31 Donghu Road, Yi Ling District, Yichang, 443100 Hubei China; 3 The RenMin Hospital, China Three Gorges University, 31 Huti Subdistrict, Xi Ling District, Yichang, 443000 Hubei China; 4 Hubei Key Laboratory of Tumor Microenvironment and Immunotherapy, China Three Gorges University, 8 Daxue Road, Xiling District, Yichang, 443002 China

**Keywords:** miR-290-295 cluster, Embryonic stem cells, Pluripotency regulation, Induced pluripotent stem cells, Tumourigenesis and senescence

## Abstract

Increasing evidence indicates that embryonic stem cell specific microRNAs (miRNAs) play an essential role in the early development of embryo. Among them, the miR-290-295 cluster is the most highly expressed in the mouse embryonic stem cells and involved in various biological processes. In this paper, we reviewed the research progress of the function of the miR-290-295 cluster in embryonic stem cells. The miR-290-295 cluster is involved in regulating embryonic stem cell pluripotency maintenance, self-renewal, and reprogramming somatic cells to an embryonic stem cell-like state. Moreover, the miR-290-295 cluster has a latent pro-survival function in embryonic stem cells and involved in tumourigenesis and senescence with a great significance. Elucidating the interaction between the miR-290-295 cluster and other modes of gene regulation will provide us new ideas on the biology of pluripotent stem cells. In the near future, the broad prospects of the miRNA cluster will be shown in the stem cell field, such as altering cell identities with high efficiency through the transient introduction of tissue-specific miRNA cluster.

## Background

microRNAs (miRNAs) are about 22 nucleotide (nt) endogenously non-coding RNAs that negatively regulate the expression of various target genes at the post-transcriptional level. Currently, in the human genome, it is reported that there are ~1500 miRNAs and each miRNA potentially modulates hundreds of target genes [1, 2]. miRNAs play important roles in various signaling pathway regulation, such as metabolism, proliferation, apoptosis, differentiation and the development of tumor.

Gene clusters are generally composed of more than two related genes which are closely located on a chromosome, and they usually share sequence similarity [3]. Increasing evidence suggests that clustered miRNA genes are generally located in a polycistron [4, 5], and co-expressed with neighboring miRNAs [6]. From the consistent expression of most miRNA clusters, it is speculated that homologous miRNA clusters may share common cis-regulatory elements, resulting in a cooperative effect for those clusters. On the other hand, for the inconsistent expression of some miRNA clusters, perhaps have different transcriptional or maturation processes. Due to functional limitations, most miRNAs are highly conserved among species. Yu et al. [7] found that partial duplications from an ancestral gene often resulted in the formation of the miRNA clusters. In addition, tandem and segmental duplications were critical for the evolution of miRNA clusters. Compared with single miRNA in regulating a complex cell signaling network, the clustered miRNAs seemed more efficient and complicated.

In 1981, Evans et al. [8] isolated mouse embryonic stem cells (mESCs) for the first time, and in 1998, Thomson et al. [9] established human ESC cell line. Since then, the research field of the stem cells has developed rapidly. With the further study in the regulation mechanism of ESCs, Shinya Yamanaka [10] successfully got the induced pluripotent stem cells (iPSCs) by introducing transcription factors Oct4, Sox2, Klf4 and c-Myc into mouse fibroblasts in 2006. Meanwhile, it has been proposed that ESCs originate from the inner cell mass of mammalian blastocysts, and hold the promise of medical applications, such as tissue engineering and stem cell therapy, which becomes a hot spot in the field of stem cell research in recent years due to their ability to self-renew and differentiate into all kinds of cell types.

There are ESC-specific miRNA clusters in human and mouse, such as miR-302 and miR-371-373 clusters in human embryonic stem cells (hESCs), miR-302 and miR-290-295 clusters in mESCs. In fact, the miR-290-295 cluster is homolog of human miR-371-373, furthermore, the miR-302 and miR-290-295 clusters share the same seed sequence, as a result, they tend to have similar function in mESCs. But miR-290-295 cluster is highly expressed in mESCs compare to the miR-302 cluster. Dgcr8 is essential for the biogenesis of miRNAs, so knocking out of Dgcr8 results in the loss of all canonical miRNAs. It has been reported that the introduction of the miR-290-295 cluster members into the *Dgcr8* ^−*/*−^ ESCs induces a highly transcriptionally homogenous population as well as wild-type ESCs [11, 12]. Furthermore, animals mostly die as embryo or infertile of female survivors when the miR-290-295 cluster is deleted [13–15], which shows the powerful features of the cluster. Therefore, it has become the focus of research. In recent years, it has been revealed that the miR-290-295 cluster plays an important role in the regulation of mESC pluripotent regulatory networks, differentiation, anti-apoptosis, as well as in the process of tumorigenesis and senescence in mouse embryonic fibroblasts. Therefore, intense research of miR-290-295 cluster will not only contribute to understanding the regulatory mechanisms in the early development of mESCs, but also help to explore the mechanisms of iPSCs and tumor regulation, so as to promote its application in the medical field.

## The structure of the miR-290-295 cluster

**Fig. 1:**
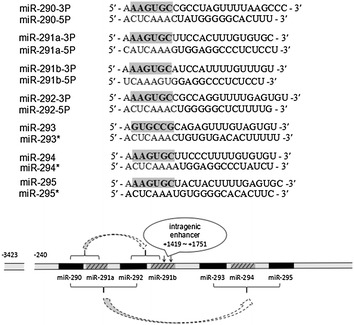
The formation process of the miR-290-295 cluster and the sequences of each member. The *bold font* are seed sequence, and the seed sequence of miR-293 is different from other members. miR-290-291a unit replication formed miR-292-291b, and then miR-290, miR-291a and miR-292 (as the same unit) replication resulted in the formation of miR-293, miR-294 and miR-295

In mESCs, the miR-290-295 cluster accounts for more than 60% of the miRNA population, however, its expression is downregulated rapidly during differentiation [13, 16]. It is a result of gradual evolution for the pre-miRNA duplications and the acquisition of new target specificities by the corresponding mature miRNAs [17]. Such duplications often form some clusters with homologous pre-miRNAs, and then co-transcribed into common primary transcripts (pri-miRNAs) [18, 19]. The variation of the seed sequences might be a reason for the homologous miRNAs to acquire the novel targets [20]. Based on sequence comparison and repeat analysis, it is proved that the miR-290-295 cluster originates from the miR-290-291a, which codes 7 miRNA precursors that give rise to 14 highly related miRNAs. The miR-290-291 unit replication forms miR-292-291b, and then the miR-290, miR-291a and miR-292 (as the same unit) replication results in the formation of miR-293, miR-294 and miR-295, finally forming the present miR-290-295 cluster [21] (Fig. 1). Within the miR-290-295 cluster, the seed sequences of ‘AAGUGC’ hexamer are found in miR-290-3p, miR-291a-3p, miR-291b-3p, miR-292-3p, miR-294, and miR-295. The other miRNAs of the miR-290-295 cluster (miR-290-5p, miR-291a-5p, miR-291b-5p, miR-292-5p, miR-293, miR-293^*^, miR-294^*^, and miR-295^*^) differing in their seed sequences, are still highly expressed in ESCs with the exception of the hardly detectable [22] minor forms of miR-293, miR-294, and miR-295 (miR-293^*^, miR-294^*^, and miR-295^*^). Tata et al. [23] identified a 332 nt intragenic enhancer (IE) region (from +1419 nt to +1751 nt) within the cluster, which was able to regulate its transcription. It was also demonstrated or predicted that numerous transcription factors regulated the cluster expression directly, such as Oct4, Sox2, Snai, Nanog and the functionally versatile CCCTC-binding factor (CTCF) [1, 23–25].

## The miR-290-295 cluster as a part of the pluripotency regulation network

ESC and iPSC self-renewals need to eliminate differentiation signal and obtain the pluripotency signal, in addition, the differentiation process trigger the closure of pluripotency procedure and the induction of lineage specification. Previously, the opinion is that regulating the pluripotent regulatory network is solely in a protein-centric approach, in recent years, however, the roles of miRNAs, especially the miR-290-295 cluster, attract more and more attention. Therefore, it will provide new insights for further study of miRNAs in the establishment and the maintenance of pluripotent regulation mechanisms of stem cells.

### The miR-290-295 cluster promotes the process of MET

**Fig. 2:**
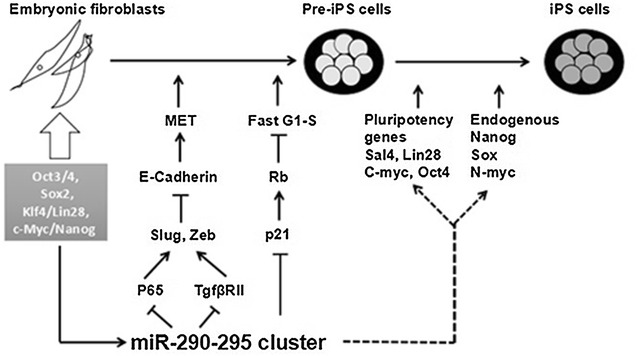
Roles of miR-290-295 cluster in enhancing somatic reprogramming. miR-290-295 cluster can enhance the reprogramming efficiency by promoting MET and cell-cycle progression in the early stage of reprogramming. In addition, it also enhances the expression of core transcription factors, such as Oct4, Sox, c-Myc, Nanog et al. in late stage

The miR-290-295 cluster promotes pluripotency by promoting mesenchymal to epithelial transition (MET). A recent research has found that MET is necessary for reprogramming of mouse fibroblasts into iPSCs [26], and the miR-290-295 cluster promotes MET by inhibiting the expression of TGF-βR2 [27, 28]. In addition, Luningschror and his co-workers [29] have demonstrated that two members of this cluster, namely miR-291b-5p and miR-293, inhibit NF-κB signaling pathway through inhibition of p65. The p65 activates the Slug and Zeb1, both of which promote the opposite process, an epithelial mesenchymal transition (EMT). Thus, the activation of NF-κB signaling pathway promotes the differentiation of stem cells by EMT, whereas the miR-290-295 cluster block it. Furthermore, Guo et al. has also confirmed that the miR-290-295 cluster suppresses EMT through repressing TGF-βR1/2 and glycogen synthase kinase 3 (GSK-3β) [30]. At the same time, Luningschror’s team provides strong evidence for the close relationship between pluripotency and epithelial phenotype. Therefore, the miR-290-295 cluster maintains pluripotency by indirectly inhibiting EMT (Fig. 2). Previously, it also has been reported that ESC-specific miRNAs participant in the inhibition of EMT and thereby involve in maintenance and induction pluripotency [27, 31, 32]. In order to better understand the relationship between pluripotency and epithelial phenotype, further studied is need.

### The miR-290-295 cluster affects the cell cycle phase distribution of ESCs

**Fig. 3:**
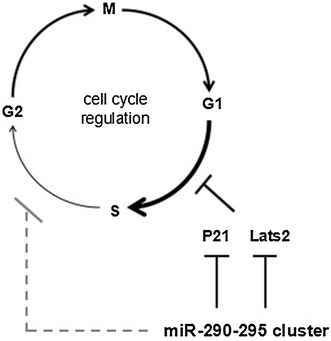
The miR-290-295 cluster affects the cell cycle phase distribution of ESC. miR-290-295 cluster can directly downregulate some cell cycle inhibitors, such as P21 and Lats2, resulting in promoting cell cycle G1/S transition. Meanwhile, it suppresses cell cycle S/G2 transition with unknown mechanism

The miR-290-295 cluster affects the cell cycle phase distribution of ESCs and maintains the pluripotency directly or indirectly. The ESCs have a unique cell cycle that the G1 phase is very short and the check point of G1/S is deficient, so that the cells quickly enter the S phase, which is similar to the cycle of cancer cells [33]. The cell cycle complex that regulates G1/S phase transition mainly includes Cyclin D/Cdk4, Cyclin D/Cdk6 and Cyclin E/Cdk2 [34], but only Cyclin E/Cdk2 complex exists in mESCs [35]. The reason is that Cyclin D1 is a direct target of miR-290-295 cluster miRNAs [36]. Downregulation of the Cyclin E/Cdk2 complex inhibitor rapidly promotes the conversion of G1/S, while the miR-290-295 cluster directly downregulates the expressions of Rb1, Rbl1, Rbl2, ARID4, p21, LATS2 and other cell cycle inhibitors result in promoting cell cycle transition [37–39]. Analogous promoting cell cycle transition effect also occurs in hESCs by miR-372 [40]. However, recent studies showed that miR-290-295 and miR-302 clusters promote the rapid G1/S transition independent of the Rb family under normal growth conditions, but just under cytostatic conditions (nutrient deprivation and cell–cell contact) their promotive G1/S transition is Rb-dependent [41]. The suppression of cell cycle inhibitors might be a molecular basis consisting of the ephemeral G1 phase of ESCs. In addition, Gonzales et al. [42] used high-throughput screening technology and found that hESCs actively resisted differentiation process and maintained their pluripotency in the S and G2 phase for the first time. Their research shows that the S and G2 phases possess an intrinsic propensity toward the pluripotent state, mediated by the ATM/ATR-CHEK2-p53 and Cyclin B1 pathways, respectively. More specifically, p53 acts to maintain the pluripotent state upon the withdrawal of self-renewal factors, and Cyclin B1 might work through TGF-β to prevent pluripotent state dissolution. Interestingly, Lichner et al. [21] found that mESCs were enriched in the S phase by high expression of miR-290-295 cluster, however, the exact mechanism is still unclear, but maybe associated with the ATM/ATR-CHEK2-p53 pathway too. All together, the miR-290-295 cluster assists stem cells to keep their pluripotency through shortening G1 and extending S phase (Fig. 3).

### The miR-290-295 cluster regulates the expression of core transcription factors

The miR-290-295 cluster establishes and maintains pluripotency of stem cells by enhancing the expression of core transcription factors. The Oct4, Sox2, Klf4/Lin28, and c-Myc/Nanog are the core transcription factors of somatic cells reprogrammed into iPSCs. Lin28 was upregulated by transfection of miR-294 into Dicer-deficient cells, but the molecular mechanism is unknown [43]. Judson et al. [44] showed the high inductive efficiency production of iPSCs with introduction of miR-290-295 cluster, and c-Myc was substituted for miR-294 successfully in somatic cell reprogramming. Thus, miR-294 is a downstream gene of c-Myc, and that miR-294 and c-Myc have some common downstream regulatory genes according to the prediction of GeneGo software, which can explain the ability of miR-294 to induce the pluripotent stem cells. The Wnt signaling pathway has been shown to be essential for maintaining pluripotency of stem cells [45, 46]. Dkk-1 has multiple roles in the cells, and the most prominent role is considered as an inhibitor of the Wnt signaling pathway [47]. Zovoilis et al. demonstrated that the Dkk-1 was a direct target of miR-294 and miR-295, and the other members of the miR-290-295 cluster controlled Wnt or Dkk-1 activation indirectly [48]. It is also confirmed that the overexpression of the miR-290-295 cluster increased c-Myc levels, which is a downstream target of the Wnt signaling pathway, while its inhibition had an opposite effect [48]. So the miR-290-295 cluster upregulates the expression of c-Myc, but the exact molecular mechanism needs to be further explored. In addition, the miR-290-295 cluster promotes the re-activation of endogenous pluripotency factor Oct3/4 by repressing NR2F2 which is a transcriptional repressor of Oct3/4 [49]. The miR-290-295 cluster also upregulates other pluripotency factors, such as N-myc, Sal4 (Fig. 2), but the specific molecular mechanism is still unclear [25, 43].

### The miR-290-295 cluster regulates the metabolism of stem cells

**Fig. 4:**
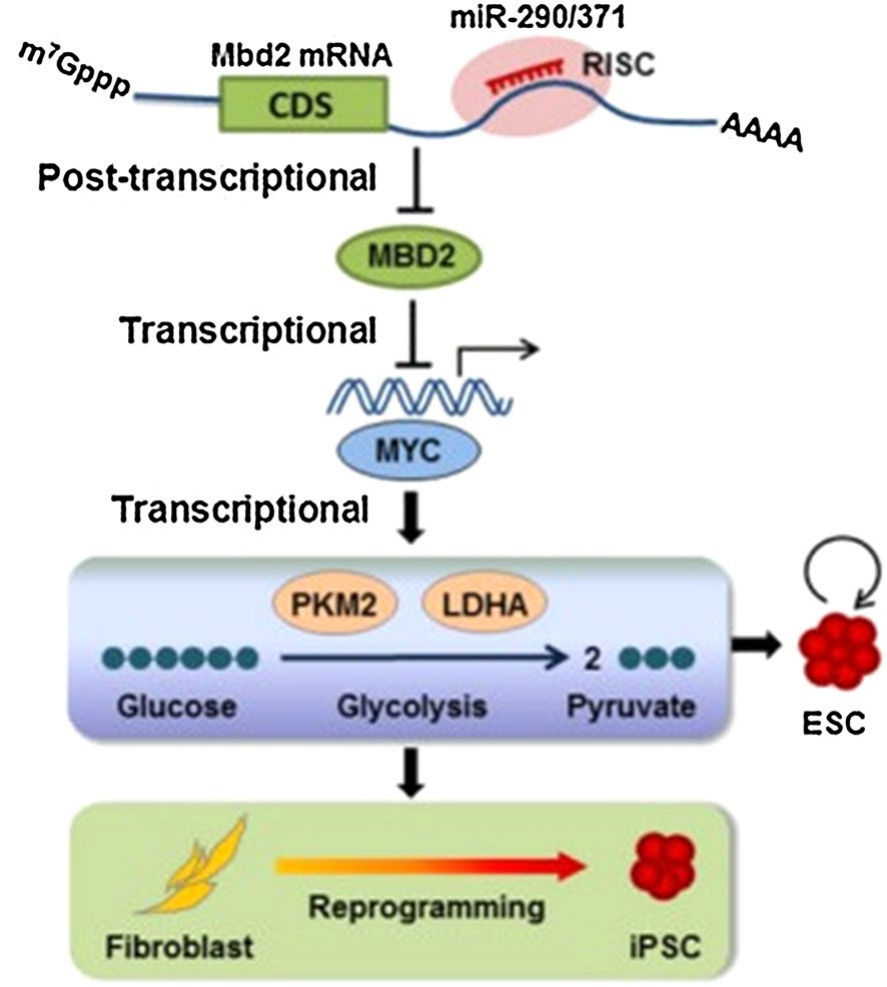
miR-371-373 cluster, homolog of human miR-290-295 cluster, stimulates the metabolic switch and reprogramming of human fibroblasts. A working model of the miR-290-Mbd2-Myc axis in regulating metabolism and reprogramming. miR-290/371 cluster post transcriptionally represses Mbd2, leading to the downregulation of MBD2 protein and reactivation of Mbd2 target gene Myc. Subsequently, Myc activates glycolysis through directly stimulating the transcription of glycolytic enzymes Pkm2 and Ldha. This regulatory circuit orchestrated by miRNAs facilitates metabolic switch in reprogramming and enhances glycolysis in ESCs (Reproduced from [58] with permission of EMBO J)

The miR-290-295 cluster regulates the metabolism of stem cells, thus maintains pluripotency. One of the important features of ESCs and iPSCs is the enhanced glycolysis, which is thought to be vital in inducing and maintaining pluripotency [50, 51]. Pluripotent stem cells use glycolysis rather than a more efficient aerobic respiration, which is similar to the Warberg effect or aerobic glycolysis in malignant tumors. In 1920, Warburg found that, compared with normal cells, cancer cells utilized a high rate of glycolysis rather than a relatively low rate of oxidative phosphorylation as energy sources even with sufficient oxygen supply [52]. More interestingly, recent studies have showed that the metabolic transition from oxidative phosphorylation to the glycolytic is also found in reprogramming from fibroblast cells to iPSCs [53–55]. In addition, forced expressions of Lin28, c-Myc and Hif1 stimulate glycolysis [53, 56, 57]. The miR-290-295 cluster stimulates glycolysis through upregulating the expression of two enzymes, namely Pkm2 and Ldha. The enzymes are essential for the induction of pluripotency during reprogramming [58]. Mechanistically, the Myc is a key player of the metabolic switch, and it is a target of Mbd2 which represses glycolysis and reprogramming. Mbd2 suppresses glycolysis through repressing the expression of Myc by binding to its promoter and methylating it. Importantly, Mbd2 is a target of the miR-290-295 cluster [58]. Similarly, miR-371-MBD2-MYC circuit promotes glycolysis and reprogramming of human fibroblasts [58] (Fig. 4).

### The miR-290-295 cluster involves in epigenetic modifications mediated by PcG proteins

**Fig. 5:**
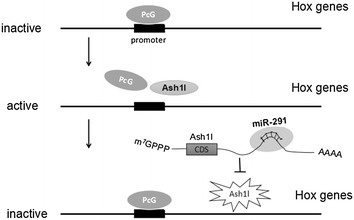
The Hox genes are associated with ESC differentiation, but they are maintained inactive in ESCs due to the action of PcG proteins. Ash1l activates Hox genes through evicting Polycomb during differentiation. miR-290-295 cluster members downregulate the expression of Ash1l to maintain pluripotency

As reported, the miR-290-295 cluster involves in Polycomb group (PcG) proteins which mediate epigenetic modifications for maintaining pluripotency. In mouse embryonic stem cells, it is characterized by the bivalent for the numerous developmentally regulated genes, with both activating and repressive histone H3 modifications at H3K4 and H3K27, respectively. Bivalency keeps genes in a poised state to enable rapid activation or stable silencing upon differentiation [59, 60]. The Hox family members belong to a series of transcription factors which are expressed by bivalent genes, and they are associated with ESC differentiation, but they are maintained inactive in ESCs due to the action of PcG proteins. PcG proteins are transcriptional repressors that regulate embryonic development and function in ESC pluripotency and iPSCs generation [61–63]. There are two Polycomb repressive complexes, PRC1 and PRC2, the former maintaining the stability of chromatin in a repressor state, and the latter playing a role in the initiation stage of transcriptional repression. PRC2 usually catalyzes the trimethylation of histone H3K27. The H3K36 methyltransferase Ash1l is one of Trithorax group proteins which are known as antagonists of Polycomb. It is recently reported that Ash1l activates Hox genes through evicting Polycomb during differentiation [64]. More interestingly, Ash1l is a target of miR-291, which was validated by using reporter assays [65]. So, miR-290-295 members regulate the targeting of PcG proteins to appropriate loci in ESCs to maintain their pluripotency (Fig. 5). In addition, a recent study has showed that miR-290-295 cluster is required for the recruiting and binding of the PRC2 core components EZH2 and SUZ12 at many bivalent promoters, for the maintenance of the bivalent state [66]. But the mechanism of miRNAs on the regulation of bivalent genes needs to be systematically investigated in the future.

## The miR-290-295 cluster also ensures the differentiation potential of pluripotent stem cells

**Fig. 6:**
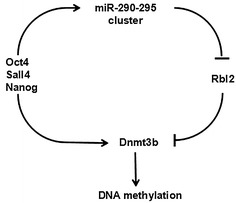
miR-290-295 cluster involved in controlling DNA methylation in the pre-implantation embryo. The feed-forward loop (FFL), that is pluripotency factors, miR-290-295 cluster, Rbl2, and Dnmt3b regulatory network, regulates DNA methylation in the pre-specified embryo and in ESCs

Epigenetic modification regulates gene expression and silence by DNA methylation, histone modification and chromatin remodeling [67]. ESCs maintain open chromatin conformations, which make it possible to switch to any type of cells [68]. Epigenetic modification plays an important role in the proliferation and differentiation of ESCs, while the miR-290-295 cluster is involved in the process. The methylation of promoter contributes to the regulation of ESC genes, while methylation deficiency may limit their developmental potential [69]. DNA methyltransferase 3b (Dnmt3b) is believed to be essential for pre-implantation development [70]. Pluripotency transcription factors, such as Oct4, Sox2, Nanog, etc. directly control the transcription of Dnmt3b, or indirectly control the expression by upregulating the expression of the miR-290-295 cluster [71]. Mechanistically, retinoblastoma-like 2 (Rbl2) is a transcription inhibitor of Dnmt3b, and Rbl2 is a direct target of miR-290-295 cluster [71, 72]. Interestingly, pluripotency transcription factors mentioned above directly control the expression of the cluster by co-occupying its promoters or enhancers. In this way, pluripotency factors, the miR-290-295 cluster, Rbl2 and Dnmt3b form a loop that regulates DNA methylation in ESCs (Fig. 6). The process of PSC differentiation requires de novo DNA methylation to silence the pluripotency program stably. In mESCs, the miR-290-295 cluster promotes the expression of Dnmt3b by targeting Rbl2, then, Dnmt3b silences Oct4, Nanog, and other factors through methylating the CpG island of them, resulting in making the normal differentiation of mESCs [72]. So the miR-290-295 cluster maintains the pluripotency of mESC, meanwhile, it prepares for the stable methylation in stem cell differentiation and full methylation during implantation. In addition, a recent study has showed that the miR-290-295/302 clusters are important regulators of naive to primed pluripotency transition [73]. These cluster members facilitate the exit of naive pluripotency in part by promoting the activity of MEK pathway and through directly repressing Akt1. The activation of MEK pathways is associated with differentiation, whereas the activation of AKT pathways is associated with pluripotency.

Intriguingly, it is known that the miR-290-295/302 clusters have also been shown to promote pluripotency in different circumstances, but how the same miRNAs possess two opposite functions remains unresolved. It is possible that the context-dependent function of the clusters in different developmental stages determines the outcome of the activity of some signaling pathways.

## The miR-290-295 cluster has the potential to promote survival of mESCs

Recent studies have shown that the miR-290-295 cluster plays an important role in cell apoptosis. Zheng et al. [74] found that the miR-290-295 cluster protected mESC cells from apoptosis during exposure to genotoxic stress through gain and loss of function studies. Further study demonstrated that the miR-290-295 cluster targeted Caspase 2 and Ei24 resulting in preventing from apoptosis of mESC gene toxicity stress through inhibiting their expression. It is the first time to link the miR-290-295 cluster with apoptosis. Ei24 promotes cell death by binding to Bcl2 [75], while Caspase 2 is an important regulatory gene in apoptosis. Subsequently, Guo et al. showed that miR-290-295/miR-302 clusters downregulated apoptosis-promoting factors Bhlhe40, Casp8, Ikbkg, Perp, on the other hand, they also upregulated the apoptosis-inhibiting factor Aven under the condition of let-7c-induced apoptosis [30]. In addition, Caspase 2 and Ei24 act as tumor suppressor genes, and their loss may contribute to tumor metastasis. For example, knockout of Ei24 in mouse fibroblasts or human breast cancer cell line, results in increasing resistance to etoposide induced apoptosis [76]. Therefore, the miR-290-295 cluster was presumed to be tumorigenic. Moreover, the miR-371-373 cluster, that is the homologue of the human miR-290-295 cluster, has been found to be highly expressed in various tumors [77–79] and to promote malignant transformation [80, 81]. Therefore, it is reasonable to speculate that this cluster has a dual role, on the one hand, it helps to protect against harmful physiological stress during development in normal cells; on the other hand, it makes cancer cells to resist the genetic toxicity of chemotherapeutic drugs.

## The miR-290-295 cluster plays a role in tumourigenesis and senescence

**Fig. 7:**
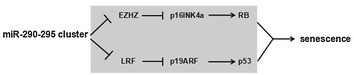
miR-290-295 cluster is causatively involved in MEF senescence. miR-290-295 cluster induces senescence through activation of INK4a/ARF locus, and the possible mechanism is to downregulate LRF with activation of p19ARF and p53, and p16INK4a up-regulation by EZH2 down-regulation

Beside the expression in stem cells, the miR-290-295 cluster is also expressed in senescent cells. The mechanisms of cell senescence is mainly divided into two categories: one way is that telomere shortening leads to double strand breaks after 50–80 population doublings, as a result, the activation of p53 leads to cell cycle arrest and senescence [82]; the other way is that some damage factors like reactive oxygen species are responsible for senescence [83]. Both of which are regulated by tumor suppressor proteins p53 and RB [84]. The major upstream regulators of p53 and RB are INK4a/ARF locus, which encodes different proteins, p16INK4a and p19ARF (mouse) or p14ARF (human), and then activates RB and p53, respectively [85]. In mouse embryonic fibroblasts (MEF), leukemia associated factor (LRF) specifically inhibits tumor suppressor p19ARF [85]. Pitto et al. [86] has clearly demonstrated that the whole miR-290-295 cluster is up-regulated in MEF in vitro, especially when the cells reach senescence. The two chromatin modifiers, the trimethylase EZH2 and the deacetylase recruiter LRF, both of which are down-regulated during MEF senescence, and are presumably responsible for removal of transcriptional silencing of the cluster by remodeling of the chromatin. Except for the miR-290-295 cluster, the expression of the p19ARF and p16INK4a are also increased, but the expression of p16INK4a is more remarkable compared with that of p19ARF [86]. As a part of PRC2 complex, EZH2 suppresses the INK4a/ARF locus, so down-regulation of EZH2 is responsible for the up-regulation of p16INK4a and p19ARF during senescence in primary fibroblast (including MEF) [87]. More interestingly, Rizzo demonstrated that the EZH2 is a target of miR-290 [85]. Further studies will determine whether the downregulation of EZH2 is responsible for the upregulation of miR-290 in senescent MEF. In addition, due to the stable mRNA level of LRF, it concludes that the reason is the post transcriptional silencing of miRNAs, and the moderator may be the increased miR-292-3P [88]. Taking together, the miR-290-295 cluster induces senescence through activation of p16INK4a/p19ARF locus. The possible mechanism is that downregulation of LRF results in the activation of p19ARF, or the inactivation of EZH2 is responsible for the upregulation of p16INK4a (Fig. 7). But recent studies have shown that the miR-290-295 cluster associates with migration and invasion of bladder cancer cells [89]. Furthermore, miR-372 and miR-373 collaborate with oncogenic Ras, and also prevent p53-driven cellular senescence by targeting LATS2 in human testicular germ cell tumor cells [81]. So there is still a lot of work to be done to determine its potential antitumor and tumorigenic effects in different genetic backgrounds.

Except for a critical role in maintaining pluripotency of stem cells, the activation of Wnt signaling pathway also occurs in various of human cancers [90, 91]. It is reported that miR-372 and miR-373 activate Wnt signaling by targeting Dkk-1, which promotes the invasive activity of tumor cells [92]. However, in hESCs, it is not reported yet whether miR-371-373 cluster maintains the pluripotency of stem cells through the activation of the Wnt signaling pathway or not. miR-373 has also been reported to promote tumor invasion and metastasis by suppression of CD44 [93]. Moreover, miR-373 drives the EMT and metastasis via the miR-373-TXNIP-HIF1α-TWIST signaling axis in breast cancer [94], but in ESCs, the miR-371-373 cluster might also maintain pluripotency by promoting MET.

## Conclusions

**Fig. 8:**
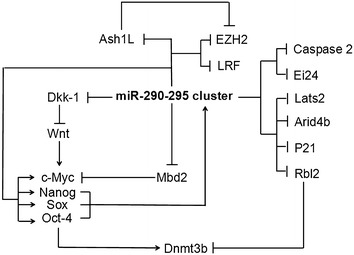
The mode pattern of the target genes regulated by miR-290-295 cluster

The miR-290-295 cluster is a cluster of important small molecule RNAs in mouse embryo, which plays an important role in cell cycle regulation, de novo DNA methylation, antiapoptosis, as well as regulation of pluripotency transcription factors. In recent years, numerous researchers were devoted to elucidate the regulation mechanism of miR-290-295 cluster, which has made some preliminary results (Fig. 8), but some challenges remained. Future studies are likely to focus on the following aspects: search for targets on a large scale. The key to study the function of a miRNA is to find the downstream target genes and understand the downstream regulatory network. There are various methods to search the target genes of miRNAs, such as computer prediction, immunoprecipitation sequencing, but these methods are not mature, and there is no method for large-scale identification of target genes, so the development of functional research of miRNAs is restricted. On the other hand, the function of the miR-290-295 cluster in mESCs needs further study. For example, the miR-290-295 cluster regulating mESC cell cycle transition is conducive to maintaining pluripotency, but the cluster also plays a role in the methylation silencing of the pluripotency factors, preparing for embryonic development. It is necessary to explore the questions about the dual function of the cluster, which dominates or regulates the function of the miR-290-295 cluster. At last, the research of the miR-290-295 cluster needs to be refined. As for many members of miR-290-295 cluster, whether there is a functional division of labor between the members, whether there is competition for target genes, and how to study the role of each member deeply and other issues need to be further studied.
